# Long Non-coding RNA MIR4435-2HG Promotes Colorectal Cancer Proliferation and Metastasis Through miR-206/YAP1 Axis

**DOI:** 10.3389/fonc.2020.00160

**Published:** 2020-02-20

**Authors:** Xinhua Dong, Zhen Yang, Hongwei Yang, Dongyan Li, Xinguang Qiu

**Affiliations:** ^1^Department of Gastroenterology, The First Affiliated Hospital of Zhengzhou University, Zhengzhou, China; ^2^Department of Thyroid Surgery, The First Affiliated Hospital of Zhengzhou University, Zhengzhou, China

**Keywords:** colorectal cancer, long non-coding RNA, MIR4435-2HG, proliferation, metastasis, YAP1

## Abstract

**Objective:** Long non-coding RNAs (lncRNAs) are critical to colorectal cancer (CRC) progression. In the current study, the objective was the exploration of the role played by lncRNA MIR4435-2HG in CRC proliferation and metastasis.

**Methods:** lncRNA MIR4435-2HG expression and its association with CRC were analyzed using database and clinical specimens. The influences exerted by MIR4435-2HG on cell proliferating process, invading process, and migrating process of CRC were identified after MIR4435-2HG knockdown. The influences exerted by MIR4435-2HG on tumor growth and metastasis were assessed *in vivo*. The underlying mechanistic associations between MIR4435-2HG, microRNA miR-206, and the transcription factor Yes-associated protein 1 (YAP1) were assessed using bioinformatics and a luciferase reporter gene assay.

**Results:** MIR4435-2HG was highly expressed in CRC tissue in contrast with that in regular tissues and displayed relations to poor prognosis. MIR4435-2HG knockdown could suppress CRC cell proliferation, invasion, and migration. Moreover, MIR4435-2HG knockdown inhibited CRC growth and liver metastasis *in vitro*. We found MIR4435-2HG knockdown reduced YAP1, CTGF, AREG, vimentin, Snail, Slug, and Twist expression but enhanced E-cadherin expression. Functionally, MIR4435-2HG acted as a competing endogenous RNA (ceRNA) to upregulate YAP1 by sponging miR-206.

**Conclusions:** MIR4435-2HG promoted CRC growth and metastasis through miR-206/YAP1 axis and is likely to play prognostic marker roles and be therapeutically targeted in CRC.

## Introduction

Colorectal cancer (CRC) refers to a top three newly identified and deadliest forms of cancer in many nations (e.g., U.S.) (1). Although great progress has been made in recent years in treating patients with CRC using radiotherapy, chemotherapy, surgical treatment, and immunotherapy (2), the prognostic process of CRC cases in late stage continues to be not favorable (3). Thus, identifying biomarkers of metastasis and therapeutic targets in CRC would facilitate favorable prognoses for patients with CRC.

Long non-coding RNAs (lncRNAs) refer to transcripts longer than 200 nucleotides without translation in protein (4). As high-throughput sequencing techniques have been leaping forward, more lncRNAs are reported participating in a range of biology-related cancer actions, covering tumor differentiation, metabolism, stemness, migration, invasion, apoptosis, and proliferation (5–7). The lncRNA MIR4435-2HG (namely, lncRNA-AWPPH, MIR4435-1HG, LINC00978, MORRBID, and AGD2) was initially identified as a prognostic biomarker for hepatocellular carcinoma (8). However, recent studies have shown lncRNA MIR4435-2HG also displays relations to cancer progression in triple-negative breast cancer (9) and osteosarcoma (10). Moreover, lncRNA MIR4435-2HG expression promotes tumor proliferation and lymph node transfer in lung cancer (11) and is a diagnosis- and prognosis-related biomarker in the plasma of gastric carcinoma cases (12). Therefore, MIR4435-2HG clearly displays relations to the production and development of several cancers.

A recent study using bioinformatics reported that lncRNA MIR4435-2HG can be regarded as an underlying biomarker to predict the prognosis of CRC (13). However, the role of LncRNA MIR4435-2HG in CRC remains unexplored. Yes-related protein 1 (YAP1) is an essential transcription factor and controls several tumor-developing influences, including cancer stemness, metastasis, invasion, migration, epithelial-mesenchymal transition (EMT), chemoresistance, and proliferation (14). In addition, rare studies are conducted on the regulation relationship between lncRNAs and YAP1. Sun et al. (15) found that lncRNA MALAT1 is induced by MIR4435-2HG and regulates angiogenesis and EMT in CRC. Moreover, lncRNA kcna3 can inhibit the progression of CRC by down-regulating YAP1 expression (16). Here, we first analyzed the relation between the clinicopathological characteristics of CRC and MIR4435-2HG. We then explored the role of MIR4435-2HG in CRC proliferation and metastasis and proved that MIR4435-2HG regulated proliferation and metastasis of CRC via miR-206-YAP1 axis. Based on these findings, MIR4435-2HG may be a supposed therapeutic target and impact the diagnosing and treating processes of CRC.

## Materials and Methods

### Clinical Specimens and Cell Culture

A total of 90 CRC tissue specimens and paired non-tumor normal tissue specimens were collected from January 2013 to January 2019 at the First Affiliated Hospital of Zhengzhou University, Zhengzhou University, Henan, China. The follow-up period was in the range (8–60 months). The median time is 52 months. All the included patients were followed up through the telephone survey. The Ethics Committee of the First Affiliated Hospital of Zhengzhou University, Zhengzhou University approved the protocol used here. Written informed consent was acquired from all recruited patients. Complete data was available for all patients and none received any antitumor treatment before surgical resection. When excised and incubated at −80°C, samples immediately received liquid nitrogen freezing process for subsequent application. American Type Culture Collection (ATCC) provided normal human colonic epithelial cell line NCM460 and the five human CRC cell lines (HT-29, SW620, LoVo, LS123 and HCT116). All cells received the growing process in Dulbecco's Modified Eagle's Medium (DMEM; Gibco) containing 10% fetal bovine serum (FBS; Gibco) at 37°C in the presence of 5% CO2.

### Real-Time Quantitative Reverse Transcription Polymerase Chain Reaction (RT-qPCR)

With TRIzol reagent (Invitrogen, Carlsbad, CA, USA) in line with the supplier's directives, RNA was extracted overall from the cells and tissues. Then, with M-MLV Reverse Transcriptase (Invitrogen), 2 μg of RNA received reverse-transcription to complementary DNA (cDNA). With SYBR® Premix Ex Taq™ II (Takara, Dalian, China), RT-qPCR was performed on a 7500 Fast RT-qPCR System. Supplementary Table 1 lists the sequences of the primers used. The small nuclear RNA U6 was an inner control over miR-206 expression, and glyceraldehyde 3-phosphate dehydrogenase (GAPDH; abs830032, Absin Bioscience Inc., Shanghai, China) was as an inner control over the expressions of the other genes analyzed. Using the 2–ΔΔCt approach, this study quantified the relative gene expression.

### Cell Proliferation and Colony Formation Assays

Cells under transfection received inoculation at a density of 2 × 10^3^ cells/well into 96-well plates and cultivation for 0, 24, 48, and 72 h to perform the Cell Counting Kit-8 (CCK-8) assays. When the various incubating times were carried out, this study introduced 10 μL of CCK-8 reagent (Dojindo Laboratories) to respective well and cultured it for an additional 2 h. With a standard microplate reader (Scientific MultiskanMK3, Thermo Scientific), the study subsequently ascertained and recorded absorbance at 450 nm. In colony formation assays, 300 transfected cells were grown into 6-well plates and cultivated for 14 d. Then clones were then fixed and stained with 0.5% crystal violet. The colonies counting and the numbers recording were conducted.

### Cell Invasion and Migration Assays

For measuring cell invasion, this study coated 24-well Transwell plates (8-μm pores; Corning, USA) using Matrigel (BD) and incubated them in a cell culture hood for 3 h at 37°C. For Transwell migration assays, only the 8-μm pore size polycarbonate membrane chamber were used. When the transfection was performed, cells received resuspending process in serum-free medium and plating process in the Transwell plates' upper chamber. The lower chamber overall contained DMEM containing 10% FBS. At 8 or 24 h post-incubation at 37°C, cells under migration on the membrane bottom surface received immobilization in methanol, staining process with 0.5% crystal violet, and counting process in five random fields under a microscope.

### Plasmid Constructs and Reagents

Short hairpin RNA (shRNA) specific for MIR4435-2HG (sh#1 or sh#2) or YAP1 (shYAP1) were used to knockdown MIR4435-2HG or YAP1, respectively, with scrambled shRNA (Ctrl) or mock-vehicle (vector) used as negative controls. The shRNA sequences of the target genes are shown in Supplementary Table 2. The shRNAs received the cloning process in lentiviral vector pLVX-shRNA1 (Clontech, USA). To establish a stable transfection cell line, pLVX-sh#1 was transfected into SW620 cells. At 10 d post-transfection, puromycin-resistant cell pools were selected. For upregulation of MIR4435-2HG, this study introduced full length MIR4435-2HG in pcDNA3.1 vectors (Invitrogen); empty plasmids were a negative control. GenePharma provided mimic and inhibitor of miR-206 and negative controls (NC mimic and NC inhibitor) to overexpress or downregulate miR-206. With Lipofectamine 2000 (Invitrogen) in line with the product protocols, this study carried out cell transfections.

### Dual-Luciferase Reporter Assay

The Dual-Luciferase® Reporter Assay System (Promega) was used in line with the producer's directives. For the assessment of the miR-206 and YAP1 interaction and miR-206 and MIR4435-2HG interaction, we generated wt-YAP1 3′-UTR-Luc, mut-YAP1 3′-UTR-Luc, wt-MIR4435-2HG 3′-UTR-Luc, and mut-MIR4435-2HG 3′-UTR-Luc reporter vectors. Synthetic oligonucleotides (Invitrogen) representing the wild-type or mutated binding sites of miR-206 in the 3′-UTR of YAP1 and MIR4435-2HG were separately cloned into the PMIR-Reporter vector (Ambion, USA). HCT116 Cells underwent co-transfection with miR-206 mimic, wt-MIR4435-2HG, mut-MIR4435-2HG, wt-YAP1, mut-YAP1, or negative control plasmids using Lipofectamine 3000 reagent (Invitrogen). At 48 h post-transfection, with the Dual-Luciferase Reporter Assay System, this study tested luciferase and renilla activities. For normalizing the luciferase active state, Renilla activity was employed.

### Western Blot Analysis

To isolate overall protein, CRC cells received lysing process with radioimmunoprecipitation assay (RIPA) buffer (89900; Thermo Scientific) containing phenylmethanesulfonyl fluoride (PMSF; Bestbio) and protease inhibitor cocktail (Bestbio, Shanghai, China). The protein samples were extracted using 10–12% sodium dodecyl sulfate-polyacrylamide gel electrophoresis (SDS-PAGE) and placed on nitrocellulose membranes, blocked with 5% skim milk at 4°C throughout the night, and subsequently cultured throughout the night with antibodies specific for YAP1 (1:1,000, ab52771, Abcam, Cambridge, MA, USA), CTGF (1:1,000, ab231824, Abcam); AREG (1:1,000, ab180722, Abcam); E-carherin (1:2,000, ab15148, Abcam); vimentin (1:2,000, ab8069, Abcam); Snail (1:1,000, ab229701, Abcam); Slug (1:1,000, ab51772, Abcam), and Twist (1:500, ab175430, Abcam). The membranes were subsequently cultivated with secondary goat anti-rabbit IgG antibody under conjugation with horseradish peroxidase (1:100, ab109489, Abcam) at 37°C for 1 h and then immersed in electro-chemiluminescence solution for imaging after which the relative protein levels were analyzed as previously described (17).

### Bioinformatical Analysis

Comparison of MIR4435-2HG mRNA expression levels in colon adenocarcinoma (COAD) and normal colon tissue were analyzed with Gene Expression Profiling Interactive Analysis (GEPIA; http://gepia.cancer-pku.cn/) (18). Prognosis based on MIR4435-2HG expression and the correlation between MIR4435-2HG and other markers were also analyzed using GEPIA. The potential MIR4435-2HG and miR-206 binding sites and miR-206 and YAP1 binding sites were predicted using StarBase v2.0 (http://starbase.sysu.edu.cn/starbase2/index.php) (19).

### Animal Models

The Institutional Animal Care and Use Committee of Zhengzhou University (Henan, China) approved all animal care, use, and euthanasia. A random comparison table was used to assure a completely randomized experiment. Accordingly, 5-week-old male nude athymic BALB/c nu/nu mice (Slack, Shanghai, China) were split to two groups in a random manner, the control group shRNA NC (Ctrl) and the experimental group shRNA-MIR4435-2HG (sh#1). To establish a subcutaneous xenotransplantation model, 3 × 10^6^ SW620 cells stably transfected with shRNA NC or sh#1 were introduced in a subcutaneous manner to the 5-week-old BALB/c nude mice. Five weeks after inoculation, the mice underwent the euthanasia, while the mass of the resulting xenografts was determined. This study recorded and ascertained tumor volume per 3 days. For establishing a mouse CRC liver metastasis orthotopic tumor model, 1 × 10^6^ SW620 cells under stable transfection with shRNA NC or sh#1 were injected into the subserosal layer of the cecum of BALB/c nude mice. At 6 week post-injection, metastatic nodules in amount of the livers of the Ctrl and sh#1 groups of mice was compared.

### Immunohistochemistry

The liver specimen of the mouse was used for immunohistochemical (IHC) staining. The procedure of IHC referred the previous study (17). The specimen was used a primary rabbit anti-YAP1 monoclonal antibody (1:1,000, ab52771, Abcam, Cambridge, MA, USA). The 3,39-diaminobenzidine (DAB) substrate kit (Vector Laboratories) was used for detecting YAP1 expression in the liver of mouse.

### Statistical Analysis

Using GraphPad Prism 7.0 software (GraphPad, Inc.), this study performed all statistics-based studies. The means ± standard deviation (SD) denotes experimental data, and all experiments were performed in triplicate. Comparison between groups was assessed by Student's *t-*test or one-way analysis of variance (ANOVA). This study performed Spearman's rank correlation analysis for evaluating variables' relations. This study is carried out Kaplan-Meier approach and log-rank experiment for analyzing the survival curves. *P* < 0.05 refers to statistics-related significance.

## Results

### Upregulated MIR4435-2HG Correlated With Poor Prognosis in CRC

**Figure 1:**
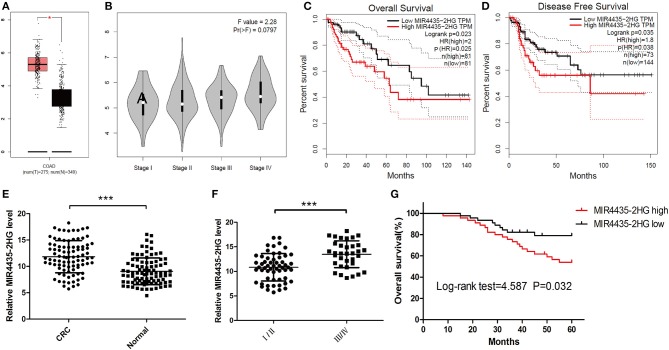
The expression and prognosis of long non-coding RNA (lncRNA) MIR4435-2HG of colorectal cancer (CRC) in The Cancer Genome Atlas (TCGA) database and clinical specimens. **(A)** Gene Expression Profiling Interactive Analysis (GEPIA) of CRC data in TCGA show lncRNA MIR4435-2HG's expression state in colon adenocarcinoma (COAD) and normal tissue. **(B)** The lncRNA MIR4435-2HG expression levels at different stages of COAD. **(C,D)** The impact of different expression levels of lncRNA MIR4435-2HG in overall survival and disease-free survival in COAD. **(E)** Comparison of lncRNA MIR4435-2HG in CRC tissues (*n* = 45) and normal tissues (*n* = 90). **(F)** LncRNA MIR4435-2HG expression at different TNM stages of CRC. **(G)** Kaplan Meier survival estimates of the correlations between lncRNA MIR4435-2HG expression and general survival of 90 cases with CRC shown in **(E)**. *P < 0.05, ***P < 0.001.

For investigating the expression profile of lncRNA MIR4435-2HG in CRC, MIR4435-2HG mRNA expression level data in The Cancer Genome Atlas (TCGA) database was analyzed first using GEPIA. The results revealed that MIR4435-2HG expression was elevated in COAD specimens in contrast to that in normal tissue (Figure 1A). MIR4435-2HG expression varied at different stages of COAD with greater MIR4435-2HG expression levels being observed in higher stages of COAD (Figure 1B). Moreover, COAD with high MIR4435-2HG expression displayed relations to disease-free survival (DFS) and poor overall survival (OS) as shown in Figures 1C,D, respectively. To further verify the results from TCGA, 90 paired CRC and regular tissues were collected from patients and the MIR4435-2HG expression levels measured. Interestingly, MIR4435-2HG expression was higher in CRC tissue compared to that in normal colon tissue (Figure 1E) and the MIR4435-2HG expression in stage III/IV tumors was significantly higher than that in stage I/II (Figure 1F). In addition, high MIR4435-2HG expression also displayed relations to poor OS in patients with CRC. Therefore, it was suggested that MIR4435-2HG was a critical cancer-promoting gene and could serve as a biomarker for the prognosis of CRC (Figure 1G).

### MIR4435-2HG Correlated to Tumor Growth and Metastasis in CRC

To delve into the relation between lncRNA MIR4435-2HG expression and clinicopathological characteristics in CRC, we compared the MIR4435-2HG expression ratio in different clinicopathological characteristics of 90 patients with CRC (Table 1). MIR4435-2HG expression was not noticeably related age, gender, differentiation grade, tumor location, lymphovascular invasion, or status of microsatellite instability (MSI)**/**mismatch repair (MMR). Interestingly, we determined that high MIR4435-2HG expression displayed relations with larger tumor size and higher tumor stage. According to the mentioned outcomes, MIR4435-2HG may play an important role in CRC growth and metastasis.

**Table 1:** Correlations between lncRNA MIR4435-2HG expression and clinicopathological characteristics in CRC.

**Parameters**	**No**	**LncRNA MIR4435-2HG**		***P*-value**
		**High (*n* = 45)**	**Low (*n* = 45)**	
Age (years)				0.520
<60	53	25	28	
≥60	37	20	17	
Gender				0.525
Male	49	23	26	
Female	41	22	19	
Differentiation grade				0.398
Well/moderate	58	26	22	
Poor/undifferentiated	32	19	23	
Tumor size (cm)				0.011
<5	44	16	28	
≥5	46	29	17	
Tumor location				0.162
Right hemicolon	24	8	16	
Left hemicolon	32	18	14	
Rectum	34	19	15	
Lymphovascular invasion				0.649
Present	62	32	30	
Absent	28	13	15	
TNM stage				0.002
I/II	56	21	35	
III/IV	34	24	10	
MSI/MMR				0.134
pMMR/ MSI-L/MSS	77	36	41	
dMMR/MSI-H	13	9	4	

*pMMR, proficient mismatch repair; MSI-L, microsatellite instability low; MSS, microsatellite stable; dMMR, deficient mismatch repair; MSI-H, microsatellite instability high*.

### MIR4435-2HG Contributed to the Growth and Proliferation of CRC Cells

**Figure 2:**
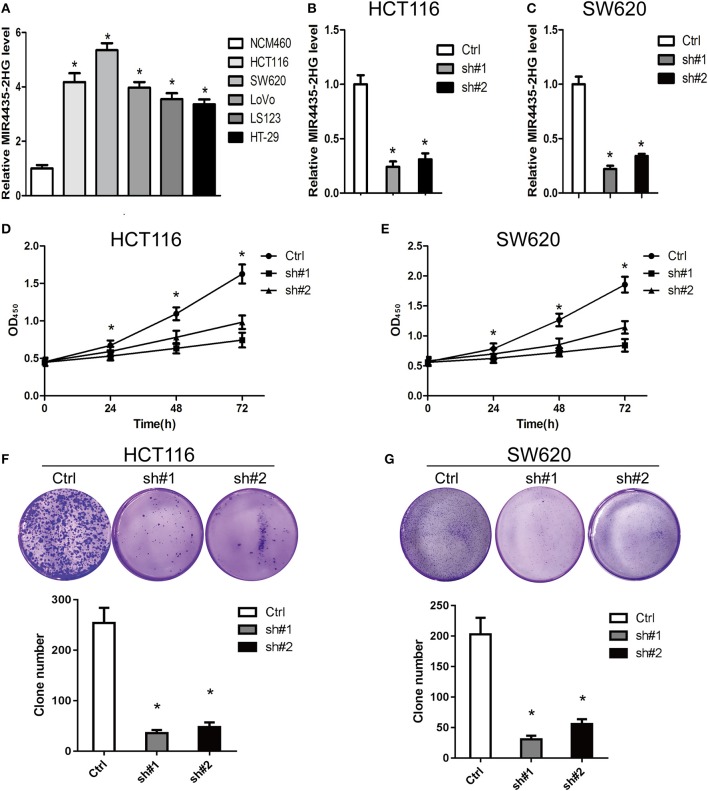
Knockdown of MIR4435-2HG inhibited growth and proliferating process of colorectal cancer (CRC) cells. **(A)** Expression analysis of MIR4435-2HG in CRC cell lines. **(B,C)** MIR4435-2HG expression was reduced by using two short hairpin RNAs (shRNAs) targeting MIR4435-2HG in HCT116 and SW620 cell lines. *P < 0.05; *n* = 3. **(D,E)** The effect of proliferation inhibition by MIR4435-2HG knockdown in HCT116 and SW620 cells in contrast to that in their respective controls was analyzed using a CCK8 assay. *P < 0.05; *n* = 3. **(F,G)** Colony formation assays were performed to evaluate cell growth following MIR4435-2HG knockdown in HCT116 and SW620 cells. *P < 0.05; *n* = 3.

For clarifying the role played by MIR4435-2HG in CRC growth, we first measure the expression states of MIR4435-2HG in different CRC cell lines. We found that MIR4435-2HG expression levels were higher in CRC cell lines HCT116, SW620, LoVo, LS123, and HT-29 in contrast with that of the regular human colon epithelial cell line NCM460 (Figure 2A).

We then knocked down MIR4435-2HG expression in HCT116 and SW620 cell lines (Figures 2B,C, respectively) and found that the suppression of MIR4435-2HG significantly inhibited HCT116 and SW620 cell proliferating process after 24 h, 48 h, and 72 h of transfection (Figures 2D,E). In addition, clone formation results also demonstrated that the growth of HCT116 and SW620 cells was significantly suppressed by the suppression of MIR4435-2HG (Figures 2F,G). Therefore, MIR4435-2HG appeared to contribute to the growth and proliferation of CRC cells, suggesting that MIR4435-2HG may be a novel target for inhibiting CRC.

### MIR4435-2HG Knockdown Inhibited the Invasion and Migration of CRC Cells *in vitro*

**Figure 3:**
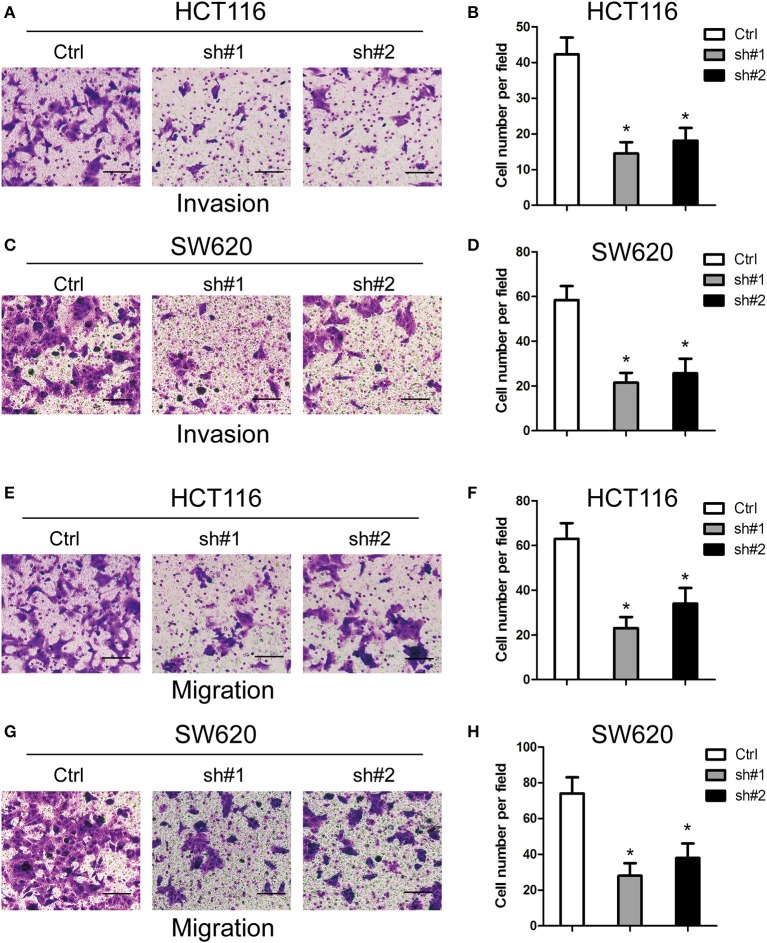
Effects of MIR4435-2HG on colorectal cancer (CRC) cells' invading and migrating processes *in vitro*. **(A–D)** Invasive potential of MIR4435-2HG knockdown in HCT116 and SW620 cells compared with that in their respective controls was analyzed using a Transwell invasion assay. *P < 0.05; *n* = 3. **(E–H)** Migratory potential of MIR4435-2HG knockdown in HCT116 and SW620 cells compared with their respective controls analyzed using a Transwell migration assay. *P < 0.05; *n* = 3.

For the investigation of how MIR4435-2HG expression related to CRC metastasis, this study ascertained the effect exerted by MIR4435-2HG expression on CRC cells' invading and migrating processes. According to Transwell invasion assays, MIR4435-2HG knockdown effectively inhibited the invasive ability of HCT116 and SW620 cells (Figures 3A–D). In addition, MIR4435-2HG knockdown hindered HCT116 and SW620 cells from migrating as indicated by it effectively reducing the number of cells in the lower chamber (Figures 3E–H). These results indicated that the MIR4435-2HG knockdown suppressed CRC cell metastasis *in vitro*.

### MIR4435-2HG Knockdown Inhibited CRC Growth and Metastasis *in vivo*

**Figure 4:**
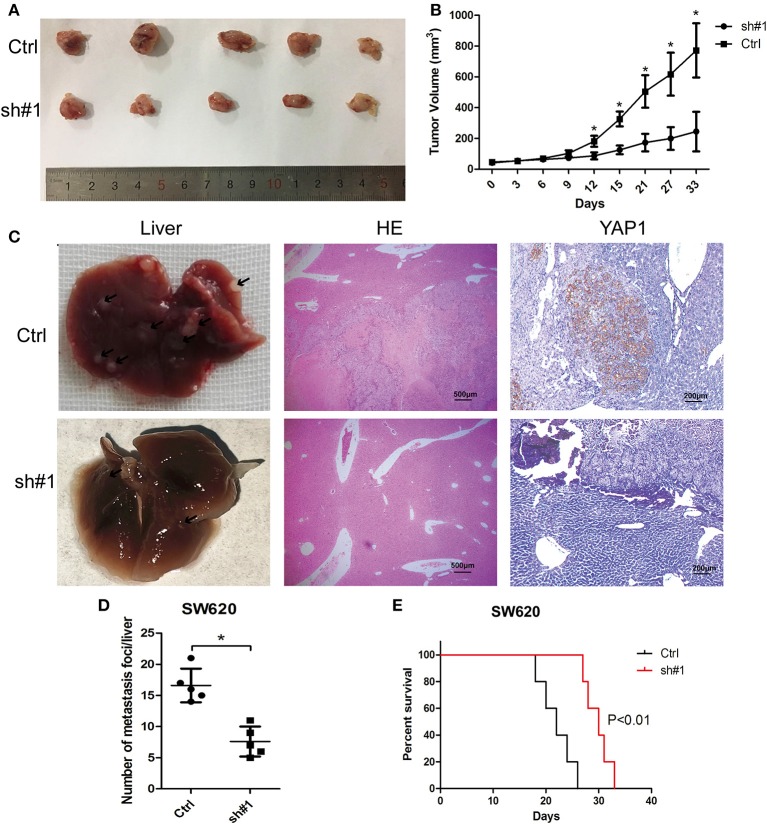
MIR4435-2HG silencing inhibited colorectal cancer (CRC) growth and metastasis *in vivo*. **(A,B)** SW620 cells where MIR4435-2HG was stably knocked down of were subcutaneously transplanted into BALB/c nude mice in a xenograft model (*n* = 5 mice per group). Two-tailed Student's *t*-test. Tumor volumes were monitored at the suggested time points in the control (Ctrl) and experimental (sh#1) groups. **(C)** Representative livers from the orthotopic mouse model of CRC, hematoxylin and eosin-stained images and immunohistochemical stained images of YAP1 show metastatic lesions and the YAP1 expression in the livers. **(D)** Mean ± SD. *P < 0.05 denotes the number of metastatic nodules in the livers; using two-tailed Student's *t*-test; *n* = 5. **(E)** Overall survival curve of mice from the orthotopic model of CRC according to the MIR4435-2HG expression (*n* = 5).

For the in-depth validation of the carcinogenic function of MIR4435-2HG *in vivo*, a subcutaneous xenotransplantation model was adopted to measure the effect of MIR4435-2HG expression on CRC growth. Tumor volumes were monitored in nude mice subcutaneously injected with MIR4435-2HG stable knockdown SW620 cells (sh#1) or control (sh-NC) transfected SW620 cells (Ctrl). The tumor volumes at the indicated time points are shown in Figures 4A,B. In general, tumor volume in the sh#1 group was noticeably inhibited in contrast with that with the Ctrl group after 12 d post-injection.

We then implemented a CRC liver metastasis orthotopic tumor model for detecting the effect of MIR4435-2HG on CRC metastasis *in vivo*. After 6 week, the number of metastatic nodules in the livers of sh#1 group was significantly less than that in the Ctrl group, the livers and metastatic nodules, as well as YAP1 expression in metastatic nodules are shown in Figures 4C,D. Moreover, the survival time of sh#1 group was significantly longer compared to that of the Ctrl group (Figure 4E). Mentioned outcomes further confirmed that MIR4435-2HG knockdown inhibited CRC growth and metastasis *in vivo*.

### MIR4435-2HG Contributed to Tumor Growth and EMT via the Hippo Signaling Pathway

**Figure 5:**
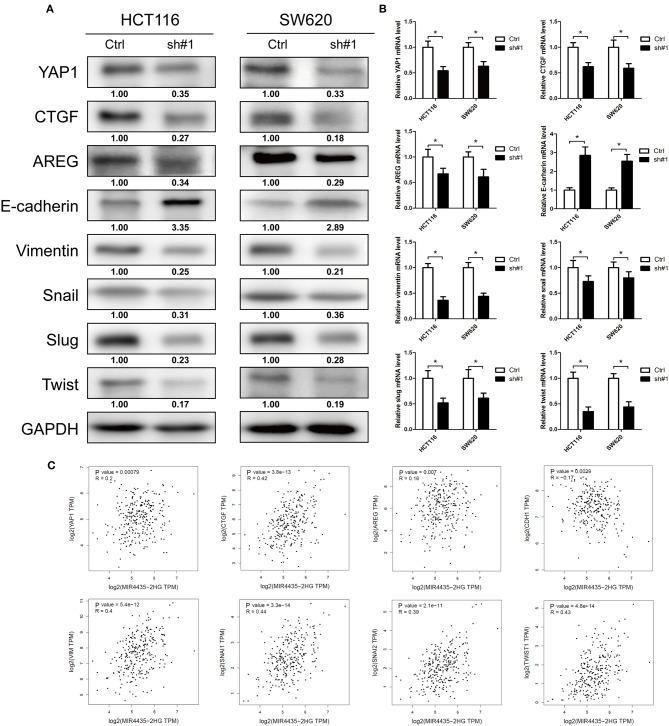
Downregulation of MIR4435-2HG inhibited tumor growth and epithelial-mesenchymal transition (EMT) via the Hippo signaling pathway. **(A)** Western blotting results show decreased protein levels of YAP1 following the knockdown of MIR4435-2HG in HCT116 and SW620 cells. Tumor growth markers CTGF and AREG expression levels were decreased after knockdown MIR4435-2HG in HCT116 and SW620 cells. Protein expression levels of mesenchymal markers vimentin, Snail, Slug, and Twist were down-regulated, and epithelial marker E-cadherin expression was increased after knockdown of MIR4435-2HG in HCT116 and SW620 cells. **(B)** Levels of YAP1, CTGF, AREG, E-cadherin, vimentin, Snail, Slug, and Twist mRNAs were measured by real-time reverse transcription polymerase chain reaction (RT-PCR). **(C)** Correlation of MIR4435-2HG with YAP1, CTGF, AREG, E-cadherin, vimentin, Snail, Slug, and Twist according to Gene Expression Profiling Interactive Analysis (GEPIA) of colorectal cancer (CRC) data in The Cancer Genome Atlas (TCGA) database. *P < 0.05.

EMT is a critical process for cancer metastasis and YAP1 is vital to activate the transcriptional programs involved in regulating EMT and tumor growth (20, 21). The relation between MIR4435-2HG and YAP1 remains unclear. Therefore, we investigated whether MIR4435-2HG regulated expression of YAP1 and its downstream proteins. In both HCT116 and SW620 cells, MIR4435-2HG knockdown decreased the expression of YAP1, CTGF, AREG, vimentin, Snail, Slug, and Twist. However, the epithelial marker E-cadherin displayed down-regulated expression in these cells (Figure 5A). We also measured mRNA expression levels of these markers using RT-qPCR. The mRNA levels following MIR4435-2HG knockdown in both HCT116 and SW620 cells were consistent with those for protein levels (Figure 5B). Lastly, we analyzed the correlation between MIR4435-2HG and the markers in COAD using information available in TCGA database (Figure 5C). As suggested from the results, MIR4435-2HG expression correlated with YAP1, CTGF, AREG, VIM, SNAI1, SNAI2, and TWIST1 expression in a positive manner. However, MIR4435-2HG negatively correlated with CDH1 expression, the gene that encodes E-cadherin. These results indicated MIR4435-2HG regulated tumor growth and EMT marker expression via the YAP1 pathway.

### MIR4435-2HG Acted as a Molecular Sponge for miR-206 and Controlled the miR-206 Target YAP1

**Figure 6:**
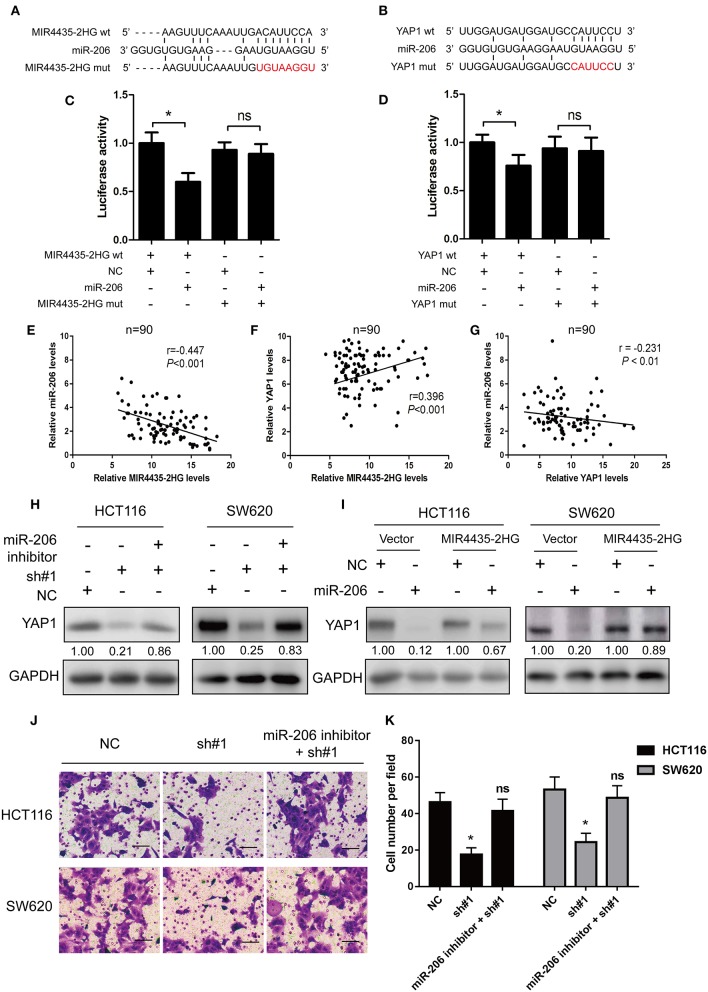
MIR4435-2HG was a molecular sponge for miR-206 and controlled the miR-206 target YAP1. **(A)** Predicted sequences of miR-206-binding sites within MIR4435-2HG. MIR4435-2HG wt, miR-206, and MIR4435-2HG mutants (mut) sequences were used in Luciferase reporter gene assays. **(B)** Estimated sequences of miR-206-binding sites within the 3′-UTRs of YAP1 and sequences of YAP1 and YAP1 3′-UTR mutants (mut) were adopted here. **(C)** Luciferase reporter gene assays were adopted for assessing the interaction between miR-206 and MIR4435-2HG in SW620 cells. **(D)** Luciferase activity in SW620 cells underwent co-transfection with miR-206 mimics and luciferase reporters containing wild-type YAP1 or mutated 3′-UTR-driven reporter constructs. **(E–G)** Correlation between MIR4435-2HG, miR-206, and YAP1 expression in CRC and normal colon specimens as detecting by real-time PCR (*n* = 90). **(H)** Western blotting assay of YAP1 protein expression in YAP1 knockdown in HCT116 and SW620 cells with and without miR-206 inhibitor. **(I)** Western blot analysis of YAP1 protein expression following expression of empty vector (NC) or MIR4435-2HG and treating process with miRNA negative control or miR-206 mimics. **(J,K)** The migration ability after MIR4435-2HG knockdown with and without miR-206 inhibitor in HCT116 and SW620 cells. *P < 0.05.

With respect to epigenetic regulation of nuclear targets, new researches proved that certain lncRNAs are competing endogenous RNAs (ceRNA) (22) and microRNA (miRNA) sponges for regulating the expressions of target genes in the cytoplasm (22). Only two studies reported MIR4435-2HG as a potential ceRNA and sponge for miR-93-3p (10) and miR-203a (23). Identifying other miRNA targets would further define the role of MIR4435-2HG in cellular functions. Note that bioinformatics study of miRNAs target recognition sequences on MIR4435-2HG and the 3′-UTR of YAP1 suggested that miR-206 complemented both the MIR4435-2HG sequence and MIR4435-2HG 3′-UTR (Figures 6A,B). To verify the mentioned result, MIR4435-2HG cDNA downstream of the luciferase gene (wt-MIR4435-2HG -Luc) was cloned. The plasmid was then co-transfected with miR-206 or a negative control. Luciferase activity was reported to be significantly reduced when miR-206 was overexpressed (Figure 6C). To determine any direct interaction between miR-206 and the MIR4435-2HG putative binding site, the miR-206 binding site was mutated using site-directed mutagenesis to generate the mut-MIR4435-2HG 3′-UTR-Luc reporter vector. In accordance with the expectation, point mutations in the tentative MIR4435-2HG abrogated the repressive effects of miR-206 (Figure 6C).

We then tested whether miR-206 targeted the YAP1 3′-UTR by performing dual-luciferase reporter assays to investigate the binding sites between YAP1 and miR-206. We found that luciferase activity was significantly reduced when wt-YAP1 3′-UTR-Luc and miR-206 underwent co-transfection in HCT116 cells. However, after mutation of the YAP1 3′-UTR and miR-206 binding sites, the repressive effect of miR-206 on YAP1 was eliminated, in contrast to what was observed with the negative control (Figure 6D).

Besides, this study also delved into the relationship between the expressions of MIR4435-2HG, miR-206, and YAP1 in the 90 CRC specimens. As shown in Figures 6E,F, MIR4435-2HG expression significantly negative correlated with miR-206 (*r* = −0.447, *P* < 0.001), while MIR4435-2HG expression displayed positive relations with YAP1 (*r* = 0.396, *P* < 0.001). For miR-206 and YAP1, we found a negative correlation between miR-206 and MIR4435-2HG expression in the 90 CRC specimens (Figure 6G).

Moreover, we wanted to determine whether MIR4435-2HG regulation of YAP1 expression was dependent on miR-206. Therefore, we co-transfected the miR-206 inhibitor and sh#1 into HCT116 and SW620 cells and evaluated the effects. The expression level of YAP1 protein was restored in contrast to that in the cells transfected with sh#1 alone (Figure 6H). The influence exerted by MIR4435-2HG expression on endogenous YAP1 both with and without exogenous miR-206 expression was assessed as well. MIR4435-2HG overexpression was reported noticeably rescuing the silencing influence exerted by miR-206 on YAP1 protein expression in HCT116 and SW620 cells (Figure 6I). Finally, the migration ability after MIR4435-2HG knockdown can be rescued by inhibiting miR-206 expression in HCT116 and SW620 cells (Figures 6J,K). On the whole, the mentioned results established that YAP1 was a target of miR-206 and that MIR4435-2HG regulated YAP1 expression in CRC by sponging miR-206.

### MIR4435-2HG Promotion of CRC Proliferation and Metastasis Was Dependent on YAP1

**Figure 7:**
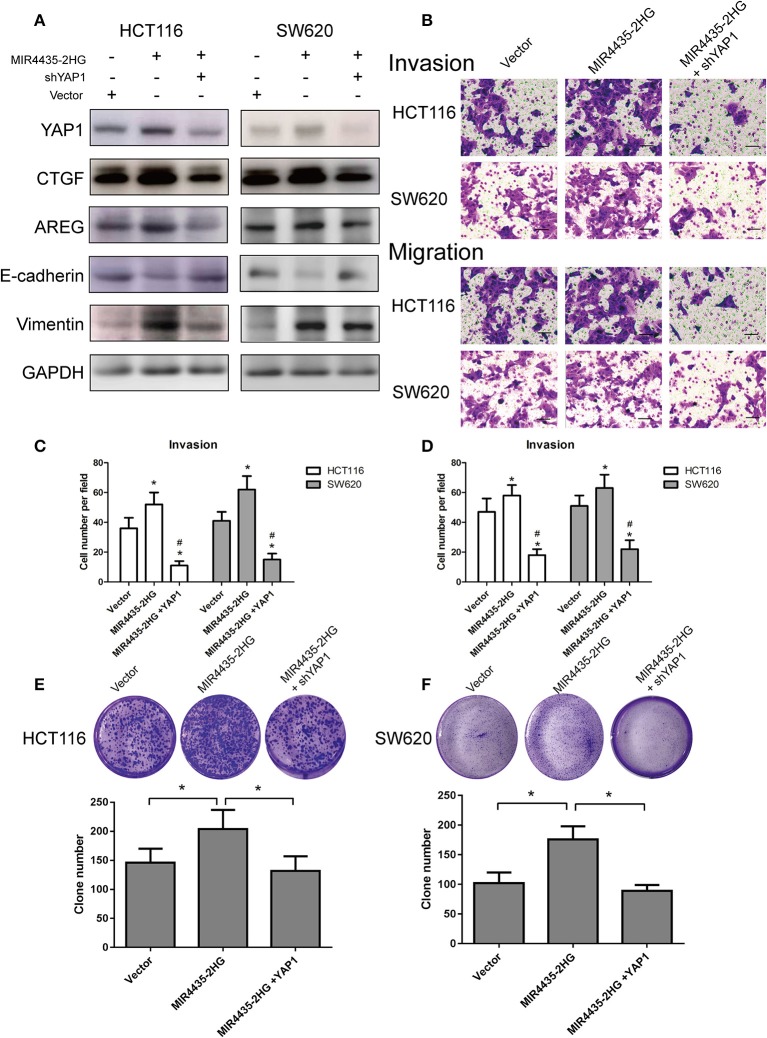
Biological function of MIR4435-2HG is determined by YAP1. **(A)** Western blotting analysis of the suggested proteins in HCT116 and SW620 cells transfected with a control vector, MIR4435-2HG, or YAP1 short hairpin RNA (shRNA). GAPDH acted as a loading control. **(B–D)** MIR4435-2HG and YAP1 shRNA vectors underwent the transfection into HCT116 and SW620 cells and cell invasion and migration were detected using Transwell invasion and migration assays. Data are shown as mean ± SD (*n* = 3). *Compared with vector *P* < 0.05; ^#^compared with MIR4435-2HG *P* < 0.05. **(E,F)** Clone formation assay*s* were used to detect the growth of HCT116 and SW620 cells after transfection with a control vector, MIR4435-2HG, or YAP1 shRNA. Mean ± SD (*n* = 3) denotes the data. *P < 0.05.

To investigate whether the MIR4435-2HG-promoted proliferation and transfer of CRC cells was dependent on a YAP1-mediated mechanism, immunoblot study was first conducted in HCT116 and SW620 cells overexpressing MIR4435-2HG with and without YAP1 knockdown. When MIR4435-2HG was overexpressed, CTGF, AREG, and vimentin expression levels were reported to be upregulated, while E-cadherin expression was decreased. We then overexpressed MIR4435-2HG and knocked down YAP1 in HCT116 and SW620 cells and observed that CTGF, AREG, and vimentin expression levels were decreased while E-cadherin expression was upregulated (Figure 7A).

We also evaluated the invasion and migration abilities of CRC cell lines when MIR4435-2HG was overexpression with or without YAP1 knockdown. Interesting, YAP1 knockdown in HCT116 and SW620 cells notably inhibited the invasion and migration abilities of MIR4435-2HG-mediated pro-metastasis (Figures 7B–D). Finally, clone formation assays also confirmed that YAP1 knockdown obviously inhibited the pro-proliferation effect mediated by MIR4435-2HG in HCT116 and SW620 cells (Figures 7E,F). Therefore, our results confirmed that CRC proliferation and metastasis promoted by MIR4435-2HG was dependent on YAP1.

## Discussion

CRC refers to a highly common gastrointestinal cancers all over the world (24). Metastasis is a common characteristic of advanced CRC and contributes the poor prognosis for patients with CRC. Though great clinical advances in therapy have been achieved, including surgery, chemotherapy, radiotherapy, and immunotherapy, 5-year survival of patients with advanced CRC remains unfavorable (25). Therefore, insights into the pathological mechanism, especially proliferation and metastasis, are imperative for the development of adequate treatments for CRC. Accumulating evidence has revealed that lncRNAs are central regulators in the pathogenesis of a diverse range of human cancers, including CRC (26). For instance, SNHG5 promotes CRC cell survival by countervailing STAU1-induced mRNA destabilization (27). Moreover, lncRNA UPAT facilitates colon tumorigenesis by inhibiting the degradation of UHRF1 (28). LncRNA PVT1-214 is an oncogene to facilitate CRC cell proliferating process and invading process by stabilizing Lin28 and interacting with miR-128 (29). Although a previous study found MIR4435-2HG an underlying biomarker to diagnose and predict the prognosis of CRC (13), the biological function of MIR4435-2HG in CRC remained unknown. Our current findings were the first to identify MIR4435-2HG as an oncogene that attributed to the proliferation and metastasis of CRC. Furthermore, our results revealed that MIR4435-2HG regulated CRC proliferation and metastasis via the miR-206/YAP1 pathway.

We initially analyzed the relation between MIR4435-2HG and the clinical characteristics of patients with CRC. TCGA data and clinical specimens were used to confirm that MIR4435-2HG expression in CRC tissue was more obvious that of normal tissue and high MIR4435-2HG expression correlated with poor prognosis. These results were consistent with a previous study (13). Interesting, our clinical data revealed that high MIR4435-2HG expression correlated with larger tumor size and more advanced TNM stage, which implied that MIR4435-2HG may be an oncogene that promoted CRC proliferation and metastasis.

Our result also confirmed that MIR4435-2HG knockdown could inhibit CRC cell growth, as well as cell invasion and migration. Recently, MIR4435-2HG was shown to be critical to cell cycle progression via mitosis (30). and that MIR4435-2HG promotes lung cancer progression through the activation of β-catenin signaling in lung and gastric cancer (11, 31). Nevertheless, the role of MIR4435-2HG in the progression of CRC is still unclear. Our results furtherly demonstrated that the loss of MIR4435-2HG leads to the reduction of cell growth and metastasis in CRC, both *in vitro* and *in vivo*. The knockdown of MIR4435-2HG not only significantly inhibited tumor growth in CRC-bearing mice, but also reduced the number of metastatic nodules in the livers of mice in a CRC liver metastasis model. Based on these results, we confirmed that MIR4435-2HG could be a therapeutic target to inhibit CRC progression.

To clarify the molecular mechanisms of MIR4435-2HG in CRC progression, we first identified whether YAP1 was regulated by MIR4435-2HG. YAP1 is a potent oncogene of the Hippo signaling pathway and is amplified in various human cancers (32). The noticeable effect exerted by Hippo pathway dysregulation and constitutive YAP1/TAZ activation on cancer cells supports them as highly attractive therapeutic targets for developing novel therapeutic approaches. However, YAP1 is regulated by multiple signals, covering those from the GPCR pathway and the mevalonate pathway and from energy stress (33). Recently, several studies have reported a relationship between YAP1 and LncRNAs in CRC. First, lncRNAs can regulate YAP1 expression. For instance, lncRNA kcna3 reduces YAP1 expression and inhibits the progression of colorectal carcinoma (16). Moreover, YAP1 is found to be a regulator of lncRNA expression in CRC, upregulates MALAT1 expression, and promotes EMT and angiogenesis in CRC (15). Pei et al. (34) suggested that the increase in YAP can facilitate carcinogenesis and transfer in human cholangiocarcinoma and rise N-cadherin expression and reduce E-cadherin expression. According to previous study (35), YAP1 enhanced TGFβ-driven Smad signaling for regulating Twist1, Slug, and Snail expressions, the critical transcriptional regulators of EMT in the atrioventricular cushion development. Moreover, YAP1 also is a crucial transcription factor for the expression of CTGF and AREG, the important gene for promoting tumorigenesis (36). In our current study, MIR4435-2HG regulated YAP1 expression in CRC and promoted CRC progression. Our results from analysis of the CRC dataset available in TCGA also confirmed that MIR4435-2HG expression significantly correlated with YAP1 expression, as well as with the expression of downstream markers of YAP1 (e.g., CTGF, AREG, E-cadherin, vimentin, Snail, Slug, and Twist). The trend of changing protein levels of these markers after MIR4435-2HG knockdown was further confirmed in HCT116 and SW620 cells. Together, these results indicated that MIR4435-2HG regulated CRC progression by regulating the YAP1 pathway.

Furthermore, we clearly demonstrated that MIR4435-2HG regulated YAP1 expression in CRC by sponging miR-206. An increasing number of studies have shown that ceRNA-base mechanisms regulated by lncRNAs are involved in several malignancies. However, only a few studies have reported the importance of miRNA MIR4435-2HG and its ceRNA mechanism (10, 23). In the current study, we found using bioinformatics analyses that miR-206 was a potential response element of MIR4435-2HG. MiR-206 was identified as a tumor suppressor gene and to inhibit tumor growth and EMT in breast and lung cancers (37, 38). The miRNA miR-206 also interacts with lncRNAs and plays a critical mechanistic role as a ceRNA. Previous studies had shown that miR-206 can be sponged with several lncRNAs in the tumor progression, such as MALAT1 (39), HOTAIR (40), and UCA1 (41). However, the relation between miR-206 and MIR4435-2HG has not been previously reported. In our current study, the dual luciferase assay results confirmed that MIR4435-2HG sponged miR-206 in CRC cells. We also are the first to identify the fact that YAP1 can be suppressed by miR-206 via interaction with the YAP1 3′-UTR. These results further confirmed that MIR4435-2HG regulated YAP1 expression by sponging miR-206. Finally, tumor-promoting effect of MIR4435-2HG could be inhibited by the knockdown of YAP1 expression. Thus, we confirmed that MIR4435-2HG-promoted CRC proliferation and metastasis was dependent on miR-206/YAP1 axis.

To summarize, we first elucidated the function and regulating system of MIR4435-2HG in CRC. As revealed from the results here, MIR4435-2HG regulated YAP1 in performing its oncogenic activities in CRC by sponging miR-206. Therefore, MIR4435-2HG has the potential of being an important prognostic marker and a therapeutic target in CRC.

## Data Availability Statement

All datasets generated for this study are included in the article/Supplementary Material.

## Ethics Statement

The studies involving human participants were reviewed and approved by the Ethics Committee of the First Affiliated Hospital of Zhengzhou University. The patients/participants provided their written informed consent to participate in this study. The animal study was reviewed and approved by Institutional Animal Care and Use Committee of Zhengzhou University.

## Author Contributions

XD conceived the project and wrote the manuscript. ZY, HY, DL participated in experiment and data analysis. XQ reviewed the manuscript.

### Conflict of Interest

The authors declare that the research was conducted in the absence of any commercial or financial relationships that could be construed as a potential conflict of interest.

## Abbreviations

lncRNAs: Long non-coding RNAs
CRC: colorectal cancer
TCGA: The Cancer Genome Atlas
YAP1: Yes-associated protein 1
ceRNA: competing endogenous RNA
EMT: epithelial-mesenchymal transition.
