# Supplementary material for: Long Non-coding RNA MIR4435-2HG Promotes Colorectal Cancer Proliferation and Metastasis Through miR-206/YAP1 Axis
Source: Front Oncol. 2020 Feb 20;10:160. doi: 10.3389/fonc.2020.00160 (PMC7044350; doi:10.3389/fonc.2020.00160)
Supplement: Supplementary file 1 [file Data_Sheet_1.docx]

Supplement Table 1. Primer sequence for RT-qPCR

| **Target genes** | **Forward primer** | **Reverse primer** |
| --- | --- | --- |
| lncRNA MIR4435-2HG | 5’-CTGGATGGTCGCTGCTTTTTA-3’ | 5’-AGGGGGATGAGTCGTGATTT-3’ |
| miR-206 | 5'-CAGATCCGATTGGAATGTAAGG-3' | 5'-TATGCTTGTTCTCGTCTCTGTGTC-3' |
| YAP1 | 5'-TAGCCCTGCGTAGCCAGTTA-3' | 5'-TCATGCTTAGTCCACTGTCTGT-3' |
| CTGF | 5'-CAGCATGGACGTTCGTCTG-3' | 5'-AACCACGGTTTGGTCCTTGG-3' |
| AREG | 5'-GTGGTGCTGTCGCTCTTGATA-3' | 5'-CCCCAGAAAATGGTTCACGCT-3' |
| E-carherin | 5'-CGAGAGCTACACGTTCACGG-3' | 5'-GGGTGTCGAGGGAAAAATAGG-3' |
| Vimentin | 5'-GACGCCATCAACACCGAGTT-3 | 5'-CTTTGTCGTTGGTTAGCTGGT-3' |
| Snail | 5'-TCGGAAGCCTAACTACAGCGA-3' | 5'-AGATGAGCATTGGCAGCGAG-3' |
| Slug | 5'-CGAACTGGACACACATACAGTG-3' | 5'-CTGAGGATCTCTGGTTGTGGT-3' |
| Twist | 5'-GTCCGCAGTCTTACGAGGAG-3' | 5'-GCTTGAGGGTCTGAATCTTGCT-3' |
| U6 | 5’-CTCGCTTCGGCAGCACA-3’ | 5’-AACGCTTCACGAATTTGCGT-3’ |
| GAPDH | 5'-ATGGAGAAGGCTGGGGCTC-3' | 5'- AAGTTGTCATGGATGACCTTG-3' |

Abbreviations: RT-qPCR, reverse transcription quantitative polymerase chain reaction; lncRNA, long non-coding RNA; YAP1, yes-associated protein 1; miR-206, microRNA-206; CTGF, **connective tissue growth factor; AREG,** amphiregulin; GAPDH, glyceraldehyde-3-phosphate dehydrogenase.

Supplement Table 2. shRNA sequences of target genes

| **Target genes** | **Sequences** |
| --- | --- |
| sh MIR4435-2HG-1 (sh#1) | 5’**-**GGTCTGGTCGGTTTCCCATTT**-**3’ |
| sh MIR4435-2HG-2 (sh#2) | 5’**-**GGAATGCAGCTGAAAGATTCC**-**3’ |
| shYAP1 | 5’**-**GGTGATACTATCAACCAAA**-**3’ |
